# Nailfold capillaroscopy in diabetes mellitus: a case of neo-angiogenesis after achieving normoglycemia

**DOI:** 10.1093/omcr/omac088

**Published:** 2022-09-26

**Authors:** Genessis Maldonado, Amala Chacko, Robert Lichtenberg, Madalina Ionescu, Carlos Rios

**Affiliations:** Internal Medicine Department, MacNeal Hospital, Berwyn, IL, USA; Internal Medicine Department, MacNeal Hospital, Berwyn, IL, USA; Internal Medicine Department, MacNeal Hospital, Berwyn, IL, USA; Endocrinology Department, MacNeal Hospital, Berwyn, IL, USA; Rheumatology Department, Universidad Espiritu Santo, Samborondón, Ecuador

## Abstract

Diabetes mellitus (DM) is a disease process characterized by a chronic hyperglycemic milieu that leads to micro and macrovascular complications, including diabetic retinopathy, diabetic nephropathy and diabetic neuropathy. During the last decade, researchers have used nail-fold capillaroscopy to study the microvascular alterations in rheumatologic diseases; however, the technology is gaining momentum in other disease processes that alter microvascular architecture. We observed a drastic improvement in the nail-fold capillary architecture in a patient with uncontrolled DM. After achieving excellent glycemic control 6 months after diagnosis, increased capillary density and evident rearrangement of the capillaries replaced the avascular areas and giant capillaries found at the time of diagnosis.

## INTRODUCTION

Nailfold capillaroscopy is a non-invasive, cost and time-efficient method to evaluate capillary architecture. The technology has long gained popularity in studying the capillary microarchitecture alterations associated with rheumatic diseases [1]. Maldonado *et al*. described an array of capillaroscopic abnormalities found in rheumatic diseases, including decreased capillary density, avascular zones, giant capillaries, ecstasies and hemorrhages. It would be only logical to extrapolate the utility of capillaroscopy to any disease process that would alter the microvascular structure, and in fact, evidence supports the hypothesis [2–4]. It is well established that persistent hyperglycemia associated with diabetes leads to alterations in capillary architecture. Vascular neogenesis in diabetic patients occurs in response to ischemia and leads to increased capillary diameter, tortuosity and decreased capillary density [5–7]. Medical literature shows that the above-mentioned capillary damages are signs of diabetes disease progression, worsening glycemic control and the manifestation of systemic complications [8].

## CASE REPORT

We present the case of a 44-year-old man with a past medical history of traumatic left eye blindness and essential hypertension, who presented to the emergency department with shortness of breath, polydipsia, decreased appetite and altered mental status for 5 days. Vitals at admission showed a heart rate of 106 beats per minute, blood pressure of 180/107 mm Hg, and respiratory rate of 28, and disoriented on physical exam. Initial laboratory workup showed a glucose level of 649 (reference range 70–99 mg/dL), arterial blood gas with a pH of 7.06 (reference range 7.35–7.45), PO2 of 143 (reference range 80–100 mm Hg), PCO2 of <19 (reference range 35–45 mm Hg), HCO3 of 3 (reference range 22–26 mmol/L), beta-hydroxybutyrate of 9.9 (reference range 0.0–0.3 mmol/L) and hbA1c of 11.9 (reference range 4.0–6.0%) (Table 1).

**Table 1:** Laboratory results

Parameters	Admission	Before discharge	After 6 months of discharge	Reference range & units
Basic metabolic panel				
Sodium	132	138	135	136–144 mmol/L
Potassium	4.7	3.8	3.8	3.3–5.1 mmol/L
Chloride	98	105	98	98–108 mmol/L
CO_2_	3	22	22	20–32 mmol/L
Anion gap	31	12	15	4–16
BUN	23	10	12	7–22 mg/dl
Creatinine	1.52	0.95	0.9	0.6–1.4 mg/dl
Glucose	659	186	118	70–100 mg/dl
Calcium	9.4	8.1	8.2	8.9–10.3 mg/dl
Estimated GFR	55	98	108	>89 mL/min/1.73 m2
B-natriuretic peptide	104			1–100 pg/ml
B-hydroxybutyrate	9.9	0.3		0.0–0.3 mmol/L
Hemoglobin A1C	11.9		5.3	4.0–6.0%
Urine analysis				
Creatinine, mg/dl	60		19	
Microalbumin	33.9		20.4	<19 mg/dl
Microalbumin/creatinine	57		32.7	<30 mg/creatinine

**Figure 1:**
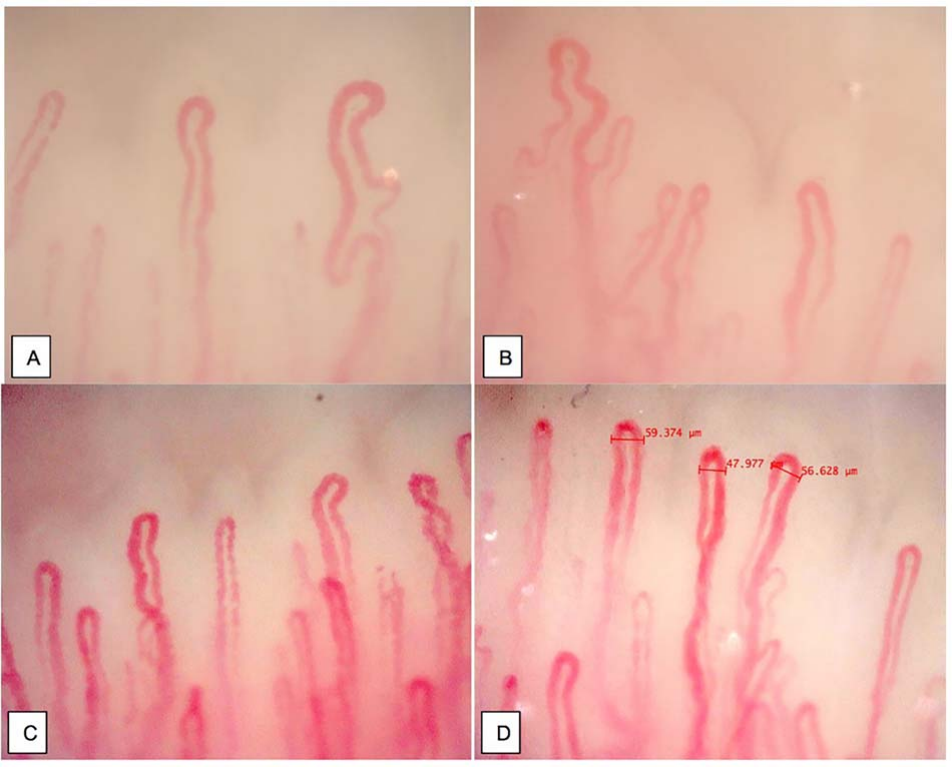
Obtained prior treatment of hyperglycemia. (**A**, **B**) Altered capillaroscopy, ramified capillaries, decreased capillary density. (**C**, **D**) Increased capillary diameters, no hemorrhages or ramified capillaries present.

We diagnosed him with diabetic ketoacidosis (DKA) in a newly diagnosed Type 2 diabetes mellitus (DM) setting and transferred him to the intensive care unit for further management. A nail-fold capillaroscopy performed at the time (Fig. 1A and B) showed altered architecture, decreased capillary density with enlarged and ramified capillaries. Rheumatology conditions were excluded, and the patient denied Raynaud’s phenomenon. No family history positive for autoimmune conditions.

After DKA resolved, hyperglycemia was corrected. Vitals signs remained stable, systolic blood pressure remained 120–130 s mmHg during admission. We educated the patient about the need for lifestyle modifications and discharged him with subcutaneous insulin, metformin and lisinopril. The HbA1c at the 6-month follow-up was 5.3%, and a repeat capillaroscopic examination revealed an increase in capillary density, and despite the dilation of capillaries, there were no hemorrhages or ramified capillaries (Fig. 1C and D). These findings intrigued us to question whether improved glycemic control would directly aid neo-angiogenesis in diabetic patients (Table 2). Patient was later referred to ophthalmology and nephrology for diabetes complication evaluation. No retinopathy was reported. Microalbuminuria improved after 6 months.

**Table 2:** Capillaroscopy description

Parameters	Admission	After 6 months	Nomenclature
Architecture	Altered	Altered	Semiquantitative scale [9]
Density	Reduced	Good	
Giant capillaries	More than 66%	Between 33–66%	Apical diameter > 50 μm
Avascular zones	Present	Improved	Loss of 2 or more contiguous capillaries
Tortuous capillaries	Present	Present	Branches that intersect like a number eight
Cross-linked capillaries	Present	Present	Capillaries with branches in an undulating, sinuous or twisted arrangement
SD-Pattern	Not present	Not present	Presence of giant capillaries, avascular zones, hemorrhages and ramified capillaries all together.

## DISCUSSION

Capillaroscopic investigations in diabetic patients started as early as 1960 [10]. Multiple studies have established that patients with poor glycemic control have a greater capillary diameter, vascular ectasias and periungual edema than healthy subjects [11–14]. The vascular damage begins with endothelial ischemia resulting in a decrement in capillary density, which leads to the release of tissue factors like VEGF, Endothelin-1, soluble thrombomodulin [15]. The compensatory neo-angiogenesis leads to ramifications and capillary dilatation [14], which unchecked can progress to the well-known microvascular complications of diabetes.

Multiple studies have established a positive correlation between nail-fold capillaroscopic pattern and glycemic control [15, 16]. In a study conducted by Lisco *et al*., the frequencies of capillary abnormalities were higher in subjects with HbA1c ≥ 7% compared to those with an HbA1c < 7% [17]. In the case described in this literature, it is evident that the capillary pattern improved considerably after achieving normalization of HbA1c. The presence of avascular zones decreased significantly, and capillary density improved.

Certain studies have pointed out an evolution time of 10 years for the appearance of ramified capillaries in diabetic patients [15, 18]. While there are contradictory data on the association of capillaroscopic findings and duration of diabetes [11, 17], studies done by Chang *et al*. and Meyer *et al*. found a positive correlation between the two [19, 20]. From Uyer *et al*. and Bracheta *et al*.’s studies, we have learned that capillaroscopy can detect microvascular changes in diabetic patients who have not yet developed clinically apparent retinopathy [10, 11].

Although abnormal microvascular architecture on capillaroscopy has been described in patients with type 2 DM, the regression of the abnormalities after normalization of the HbA1c warrants further studies; this is the first case reported. If there is a definite correlation between strict glycemic control and normalization of microvascular architecture, the scope of capillaroscopy can be broadened to screening, monitoring and progression of microvascular complications of DM.

## CONCLUSION

Capillaroscopy is an excellent diagnostic tool for evaluating disease progression and development of complications in DM. It is a non-invasive, portable and cost-effective study with reproducible results. Although capillaroscopic findings are operator-dependent and could interfere with the interpretation, a validated report could make the interpretation more homogeneous and standardized [9]. Tortuosity, bushy capillary, neoformation, bizarre capillary, microhemorrhage, capillary ectasia and aneurysm are the most common capillaroscopic findings in diabetic patients [10, 19]. Early detection of capillaroscopic abnormalities could serve as a prognostic indicator of diabetic retinopathy [10] and pre-diabetes [17], giving us a preventive edge over major diabetic complications like blindness and chronic kidney diseases that directly correlate to the functional quality of life.

## AUTHORS’ CONTRIBUTION

Genessis Maldonado was responsible for case report conception and design. Genessis Maldonado and Amala Chacko wrote the manuscript draft. Robert Lichtenberg, Madalina Ionescu and Carlos Rios provided critical revision.

## DATA AVAILABILITY

The authors confirm that the data supporting the findings of this study are available within the article.

## CONSENT

The patient signed informed consent to release the information mentioned in the case report.

## CONFLICTS OF INTEREST

The authors declare no conflicts of interest.

## FUNDING

We did not receive financial aid from any organization. The authors are part of the Internal Medicine and Endocrinology Department at Loyola MacNeal Hospital and the Rheumatology department at Universidad Espiritu Santo.
